# MAP1B Interaction with the FW Domain of the Autophagic Receptor Nbr1 Facilitates Its Association to the Microtubule Network

**DOI:** 10.1155/2012/208014

**Published:** 2012-05-10

**Authors:** Katie Marchbank, Sarah Waters, Roland G. Roberts, Ellen Solomon, Caroline A. Whitehouse

**Affiliations:** ^1^Department of Medical and Molecular Genetics, Kings College London, London SE1 9RT, UK; ^2^The Randall Division for Cell and Molecular Biophysics and Cardiovascular Division, British Heart Foundation Centre of Research Excellence, King's College London, London SE1 1UL, UK

## Abstract

Selective autophagy is a process whereby specific targeted cargo proteins, aggregates, or organelles are sequestered into double-membrane-bound phagophores before fusion with the lysosome for protein degradation. It has been demonstrated that the microtubule network is important for the formation and movement of autophagosomes. Nbr1 is a selective cargo receptor that through its interaction with LC3 recruits ubiquitinated proteins for autophagic degradation. This study demonstrates an interaction between the evolutionarily conserved FW domain of Nbr1 with the microtubule-associated protein MAP1B. Upon autophagy induction, MAP1B localisation is focused into discrete vesicles with Nbr1. This colocalisation is dependent upon an intact microtubule network as depolymerisation by nocodazole treatment abolishes starvation-induced MAP1B recruitment to these vesicles. MAP1B is not recruited to autophagosomes for protein degradation as blockage of lysosomal acidification does not result in significant increased MAP1B protein levels. However, the protein levels of phosphorylated MAP1B are significantly increased upon blockage of autophagic degradation. This is the first evidence that links the ubiquitin receptor Nbr1, which shuttles ubiquitinated proteins to be degraded by autophagy, to the microtubule network.

## 1. Introduction

Cellular turnover of damaged and misfolded proteins is mediated by two main degradation pathways; macroautophagy (hereafter referred to as autophagy) and the ubiquitin proteasome system (UPS). The UPS targets soluble, cytosolic proteins to the proteasome where they are degraded. Proteins targeted for degradation are covalently modified by the small, highly conserved, ubiquitously expressed protein ubiquitin. Ubiquitin can form chains at all seven lysine residues and typically, chains of four or more ubiquitin molecules are required for the targeting of proteins to the proteasome [1]. However, misfolded proteins can form large aggregates which render them resistant to proteasomal degradation [2]. Autophagy is an evolutionary conserved catabolic process that serves to deliver large polyubiquitinated protein aggregates and whole organelles to the lysosome for degradation [3]. A block in this process can cause the accumulation of ubiquitinated protein aggregates and ultimately cell death [4].

Autophagy requires the coordinated action of 35 to date autophagy-related genes (ATG) that mediate the formation of the double-membrane bound autophagosome which encloses a portion of the cytoplasm and delivers it to the lysosome [5, 6]. There are two ubiquitin-like conjugation systems that are required for autophagosomal formation. The Atg12-Atg5-Atg16L complex is important for elongation of the isolation membrane [7] whilst Atg8/LC3, covalently attached to phosphatidylethanolamine (PE) is essential for autophagosome biogenesis [8]. LC3 is often used as a marker for autophagosomes and has been shown to bind and stabilise microtubules [9, 10]. The microtubule network is important for autophagosomal formation [11, 12]; however, its requirement for fusion of autophagosomes with lysosomes is still unclear [11–13]. Roles for distinct populations of microtubules have also been proposed whereby labile microtubules specifically recruit markers of the isolation membrane such as Atg5, Atg12, and LC3 to sites of autophagosomal formation whereas stable microtubules facilitate the movement of mature autophagosomes [14].

Recent evidence demonstrates that autophagy can be a selective process, whereby single proteins and cellular structures such as aggregates and organelles can be specifically targeted to autophagosomes [15, 16], but the molecular mechanism of cargo recognition is poorly understood. Recently autophagic receptors have been described which include the structurally similar proteins p62 and NBR1, as well as the TBK1 adaptor NDP52 [17–19]. These receptors are thought to bind to polyubiquitinated proteins via their C-terminal-ubiquitin-associated (UBA/UBZ) domains and sort them to sites of autophagosomal formation via their interaction with LC3 [20, 21]. Both NBR1 and p62 colocalise with ubiquitin in Mallory bodies in the liver of patients with alcoholic steatohepatitis [18] and accumulate with ubiquitin in muscle fibres of sporadic inclusion-body myositis [22]. In contrast to p62, NBR1 has not been extensively studied, however growing evidence has implicated it in a diverse range of biological functions. NBR1 interacts with the giant sarcomeric protein titin and is part of a signalling complex that regulates muscle gene expression [23]. A genetically modified mouse model expressing a C-terminally truncated form of Nbr1 identified a role for Nbr1 in bone remodelling whilst a T-cell-specific knock-out of full length Nbr1 has implicated NBR1 as a mediator of T-cell differentiation and allergic inflammation [24, 25]. NBR1 has also recently been shown to direct autophagic degradation of mid-body derivatives, independent of p62 [26]. Additionally, NBR1 inhibits receptor tyrosine kinase (RTK) degradation by trapping the receptor at the cell surface [27] and via its interaction with SPRED2, mediates the lysosomal degradation of activated receptors and the attenuation of fibroblast growth factor (FGF) signalling [28]. Identification of other protein interactors of NBR1 such as calcium- and integrin-binding protein (CIB) and fasciculation and elongation protein zeta-1 (FEZ1) [29] have suggested additional roles for NBR1 in cardiac dysfunction [30] and neuronal development, respectively [31]. It has been shown that both NBR1 and p62 are recruited to autophagosomal formation sites independent of LC3; however, the mechanism is unclear [32].

In this paper, we identify NBR1 as an interaction partner of the microtubule-associated protein MAP1B. This occurs via the evolutionarily conserved FW domain. We show that whilst MAP1B is not itself a substrate for autophagosomal protein degradation, the phosphorylated form of MAP1B is stabilised by lysosomal inhibition. We propose that this interaction provides a mechanism by which NBR1 is targeted to the microtubule network to promote degradation of proteins via the autophagosome.

## 2. Materials and Methods

### 2.1. Bioinformatics

BioEdit was used to curate sequences and compile alignments. BLAST was used on various databases to identify FW-like sequences from animal, plant, fungal, protist, bacterial, and metagenome sequences. Phyre was used for structural predictions.

### 2.2. Primary Antibodies and Constructs

For western blot analysis and immunofluorescence the following antibodies were used: polyclonal anti-myc (A14, Santa Cruz), monoclonal anti-HA (Roche), monoclonal anti-myc (9E10, Santa Cruz), and polyclonal anti-MAP1B-HC (kindly provided by Prof. Gordon-Weeks, King's College London [33, 34], polyclonal anti-MAP1B (N19, Santa Cruz), polyclonal anti-MAP1B (C20, Santa Cruz), polyclonal anti-pThr1265-MAP1B (Novus Biologicals), monoclonal anti-Nbr1 (Abcam), monoclonal anti-p62 (Abnova), and polyclonal anti-p62 (kindly provided by Prof. Gautel, King's College London), polyclonal anti-ULK1 (Sigma), polyclonal anti-ubiquitin (Dako), polyclonal anti-EEA1 (Cell Signaling), polyclonal anti *β*-actin (Abcam), and monoclonal anti-His (Novagen).

Yeast two-hybrid bait for Nbr1 was amplified by PCR and cloned into pGBKT7 (Nbr1 aa346-498) (Clontech). Full length Nbr1 was cloned into pHM6 (Roche) and MAP1B aa2216- 2464 was cloned into pcDNA3.1 (Invitrogen) for the coimmunoprecipitation assay. Nbr1 aa346-498 was cloned into pGEX2T (GE Healthcare) for the GST-binding assay and MAP1B aa2227-2464 was cloned into PET6H (a modified version of pET11d-Novagen) for the recombinant binding assay.

### 2.3. Yeast-2-Hybrid

Yeast strain Y187 was transformed with the Nbr1 bait construct and mated with a pretransformed (yeast strain AH109) mouse neonatal calvarial cDNA library kindly supplied by Prof. Ikramuddin Aukhil, University of Florida. Resulting colonies were screened by HIS3 reporter gene activity, replated three times and inserts were sequenced. Y187 transformed with pGBKT7 Nbr1 aa346-498 was mated with yeast strain AH109 expressing the library MAP1B clone pGADT7 MAP1B aa2238-2465 and plated onto SD medium lacking leucine, tryptophan, histidine and adenine and were cultured at 30°C to verify the interaction.

### 2.4. Coimmunoprecipitation

COS7 cells were cotransfected with HA-Nbr1 and MAP1B-myc and after 48 hours, lysed in IP buffer (50 mM Tris pH 7.5, 150 mM NaCl, 0.5% NP-40, supplemented with protease and phosphatase inhibitors (Roche)), and cell lysates incubated with rabbit polyclonal anti-myc antibody overnight at 4°C. Protein A beads (Millipore) were then added to the lysates for a further 2 hours, beads were then washed three times in IP buffer. Proteins retained on the beads were separated by SDS-PAGE and transferred onto a nitrocellulose membrane following standard procedures. Blots were probed with mouse monoclonal anti-myc and rat monoclonal anti-HA antibodies and subsequently with a secondary antibody (HRP-conjugated anti-mouse or anti-rat, Dako, Abcam). Detection was performed by ECL (GE Healthcare).

### 2.5. Bacterial Expression of Fusion Proteins

Nbr1 aa346-498 fused to GST and GST alone were expressed in Bl21(DE3) bacterial cells and proteins purified by glutathione affinity chromatography as previously described [35]. MAP1B aa2227-2464 fused to His6 was also expressed in Bl21(DE3) bacterial cells and purified in the presence of urea. Briefly, bacterial cells expressing His_6_-MAP1B aa2227-2464 were lysed in lysis buffer (100 mM NaH_2_PO_4_, 10 mM Tris pH 8, 6 M Urea, 5 mM Imidazole pH 8, supplemented with EDTA-free protease inhibitors (Roche)). The sample was sonicated, centrifuged, and the supernatant was incubated with Ni Sepharose 6 fast flow beads (Amersham Biosciences) for 2 hours at 4°C. Beads were then washed in low Imidazole elution buffer (100 mM NaH_2_PO_4_, 10 mM Tris pH 8, 6 M Urea, 20 mM Imidazole pH 8, supplemented with EDTA free protease inhibitors (Roche)) and bound proteins eluted from the beads using high Imidazole elution buffer (100 mM NaH_2_PO_4_, 10 mM Tris pH 8, 6 M Urea, 250 mM Imidazole pH 8, supplemented with EDTA-free protease inhibitors (Roche)). The resulting purified His-tagged protein was dialysed into 50 mM Tris pH 7.5, 150 mM NaCl and used in the GST pull-down assay.

### 2.6. GST Pull-Down Assays

COS7 cells were transfected with a MAP1B-myc construct and after 48 hours expression, lysed in IP buffer (as above), and lysates incubated with beads coupled with either GST-Nbr1 aa346-498 or GST alone for 2 hours at 4°C. Following incubation, beads were washed three times in IP wash buffer (50 mM Tris pH 7.5, 200 mM NaCl, 0.5% NP-40, supplemented with protease and phosphatase inhibitors (Roche)) and proteins retained on the beads were analysed by western blotting as described above, using the monoclonal anti-myc antibody, 9E10. Alternatively purified GST or GST-Nbr1 aa346-498 attached to glutathione agarose beads (Sigma) was incubated in IP buffer (50 mM Tris pH 7.5, 150 mM NaCl, 0.5% NP-40, supplemented with protease and phosphatase inhibitors (Roche)) with purified His_6_-MAP1B aa2227-2464 for 2 hours at 4°C. Following incubation, beads were washed three times in IP wash buffer (50 mM Tris pH 7.5, 200 mM NaCl, 0.5% NP-40), and proteins retained on the beads were separated by SDS-PAGE and analysed by western blotting as described above, using the anti-His tag monoclonal antibody.

### 2.7. Cell Culture, Treatments, Transfection and Immunostaining

COS7 cells were cultured in DMEM/10% FCS by standard protocols and transfected using Fugene 6 (Roche). Cells were lysed 48 hours later in 200 *μ*L IP buffer for pull-down and coimmunoprecipitation assays. For immunostaining PC12 cells were cultured on coverslips in DMEM/10% FCS, treated with DMSO or Bafilomycin A1 (Sigma) for 4 or 8 hours, or starved in Hanks-Balanced Salt Solution (Sigma) for 4 hours or treated with 5 *μ*g/mL nocodazole (Sigma) for 30 minutes before or after 2 hours starvation and fixed in 4% paraformaldehyde/PBS for 10 minutes. Cells were then permeabilised in 0.1% Triton X100/PBS and incubated consecutively with primary and secondary antibodies (Dako) for one hour each prior to mounting. Cells were imaged using a Zeiss LSM 510 confocal microscope in sequential scanning mode with a Plan-Apochromat 63 x/1.4 Oil DIC objective. Quantification of MAP1B/Nbr1 colocalisation was performed using Zeiss ZEN2010 software, data represent mean ± SEM of 22 images.

## 3. Results

### 3.1. The Predicted Structure and Evolution of Nbr1 FW Domain

We used BLAST-based searches to acquire NBR1-related sequences from multiple available genomic and transcriptomic sources across a broad range of eukaryotes. These identified a region of pronounced conservation of 105 amino acids (residues 374–478 of human NBR1) which is recognisable in the single NBR1 orthologue found in most eukaryotes but is absent from p62. This novel domain has been named the NBR1 domain [36] and FW domain by Terje Johansen's group [37] after its four strikingly conserved tryptophan residues, and we will use FW nomenclature here for clarity.

**Figure 1:**
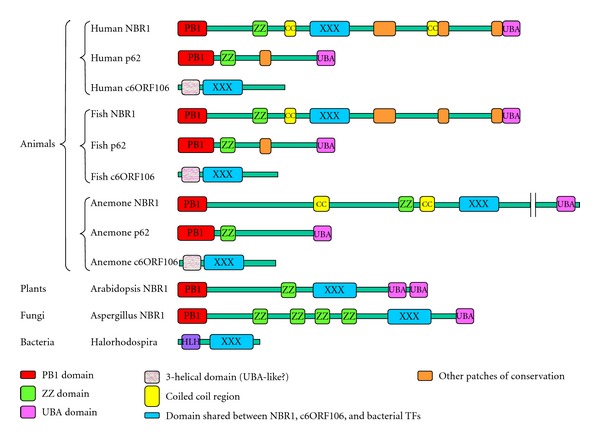
Schematic representation of the commonest members of the FW-containing protein families- the NBR1 proteins found in almost all eukaryotes, the c6ORF106 proteins found in almost all metazoans and the XRE-FW proteins found in some bacteria. The metazoan p62 family is also included to show its relationship to NBR1. Proteins are drawn to scale. A key to the domains appears at the bottom of the figure.

Single NBR1 orthologues were found in all animals, most plants, most fungi (though notably not *Saccharomyces cerevisiae*) and some single-celled eukaryotes (such as *Dictyostelium discoideum*). In each case the NBR1-like molecule possessed an N-terminal PB1 domain, one (animals, plants) or more (fungi) ZZ domains, an FW domain, and a C-terminal UBA domain (Figure 1).

The FW domain was also found in a second, otherwise unrelated animal protein. As the human version has been named c6ORF106, we will use this name. Single c6ORF106 orthologues are found in all animal species examined, plus the single-celled metazoan sister-group choanoflagellates. No c6ORF106 orthologues were found in any other organisms. The proteins tend to be small (the human c6ORF106 is 298 amino acids long), comprising a universally conserved N-terminal *α*-helical domain of ~70–80 amino acids, then the FW domain and finally a poorly structured and variable length C-terminal region (Figure 1).

Intriguingly, FW domains were also found in a wide range of eubacteria. The eubacterial FW-containing proteins are strikingly diverse in domain structure, with the only common theme being that the FW domain tends to be very close to the C-terminus. Although most eubacterial genomes do not encode an FW domain-containing protein, we find that it is broadly distributed across eubacterial clades (*γ*-proteobacteria, chloroflexi, actinobacteria, and several unclassified metagenomes). In several of the bacterial proteins (*Halorhodospira halophila, Methylomonas methanica, Kribbella flavida, Variovorax paradoxus,* and one from a fresh water environmental metagenome), the FW domain appears immediately C-terminal to a robustly predicted “helix-turn-helix” DNA-binding motif of the XRE family. We call these XRE-FW proteins. XRE domains tend to appear either alone or with multimerisation domains (as in the *Bacillus subtilis* repressor of sporulation and biofilm formation, SinR and the bacteriophage repressors CI and Cro). This juxtaposition raises the possibility that the FW domain might mediate homo- and/or heterodimerisation or could bind a small signalling molecule. Some other eubacterial FW proteins consist solely of two tandem FW domains and little else (e.g., that from *Coprococcus*), while others contain transmembrane domains (e.g., that from *Streptomyces* sp.). This structural diversity suggests that the FW domain has a generically useful function that has been exploited in many ways.

**Figure 2:**
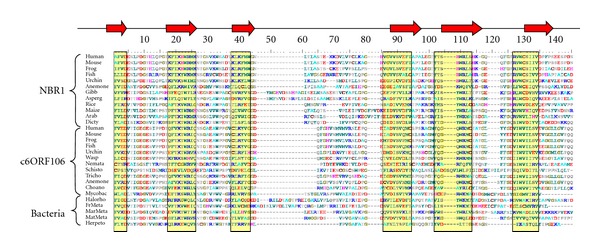
Alignment of NBR1, c60RFI06 and eubacterial FW domain sequences. Yellow boxes and red arrows indicate *β*-strands predicted by Phyre. The amino acids are colour coded as follows; red-positively charged, dark blue-negatively charged, grey-non-charged polar, dark green-aliphatic and aromatic, cyan-alanine, brown-cysteine, magenta-histidine, gold-glycine. Brackets at left indicate broad origin of FW domains (according to gross structure of host protein or phylogenetic affinity). Species are indicated as follows: Human—*Homo sapiens*; Mouse—*Mus musculus*; Frog—*Xenopus tropicalis*; Fish—*Danio rerio*; Urchin—*Strongylocentrotus purpuratus*; Anemone—*Nematostella vectensis*; Gibb—*Gibberella zeae*; Asperg—*Aspergillus nidulans*; Rice—*Oryza sativa*; Maize—*Zea mays*; Arab—*Arabidopsis thaliana*; Dicty—*Dictyostelium discoideum*; Wasp—*Nasonia vitripennis*; Nemato—*Caenorhabditis elegans*; Schisto—*Schistosoma mansoni*; Tricho—*Trichoplax adhaerens*; Choano—*Monosiga brevicollis*; Mycobac—*Mycobacterium* sp. MCS; Halorho—*Halorhodospira halophila*; FrMeta—Fresh water metagenome; MarMeta—Marine metagenome; MatMeta—Mat metagenome; Herpeto—*Herpetosiphon* sp.

We used Phyre to predict the secondary structure of all FW domains separately. This robustly predicted the same alignable structural features in every sequence, regardless of sequence divergence (Figure 2). Thus we feel that we are able to say with some confidence that the FW domain consists of two sets of three *β*-strands separated by a central unstructured region of more variable length. Striking sequence features include four almost invariant tryptophan residues, which lie in the middle of strand *β*2, in the linker between *β*2 and *β*3, and in the middle of strands *β*5 and *β*6. These give the domain its name and are only rarely replaced by other aromatic residues. There are also several invariant glycines and prolines in some of the unstructured linkers. It is conceivable that the domain folds into a sandwich of two three-strand *β*-sheets with the tryptophans projecting into the hydrophobic core.

Phylogenetic analysis showed that all NBR1 FW domains clustered together, as did all c6ORF106 FW domains. Two FW sequences from metagenomic sources clustered with NBR1 sequences; one of these had a C-terminal UBA domain, and we assume that these are from eukaryotic species in the environmental metagenome sources. The reproducible monophyletic clustering of bacterial FW domains (to the exclusion of eukaryotic NBR1 and c6ORF106 sequences) argues against multiple eukaryote-to-prokaryote horizontal gene transfer events and suggests that the FW domain may be ancient, predating the split between eukaryotic and eubacterial domains.

### 3.2. Identification of the FW Domain of Nbr1 as an Interaction Partner of Microtubule-Associated Protein MAP1B

**Figure 3:**
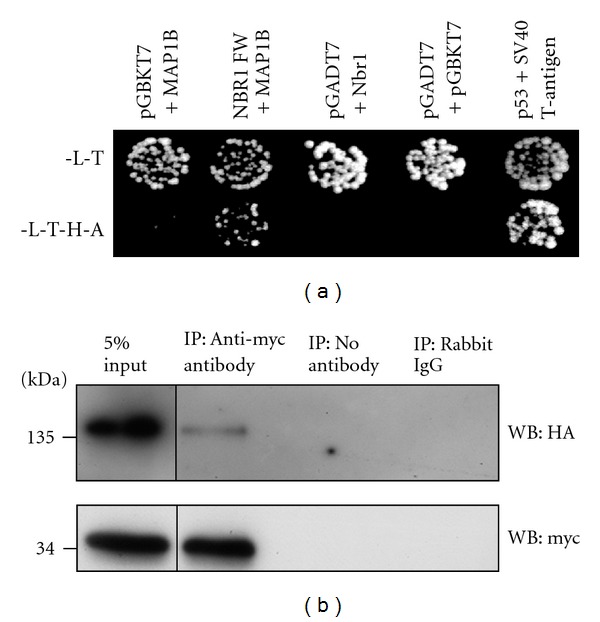
Nbr1 interacts with MAP1B *in vivo*. (a) Identification of Nbr1 as an interaction partner of MAP1B-LC1. Yeast-2-hybrid retransformation assay confirming the interaction between the FW domain of Nbr1 (aa346-498) and the light chain of MAP1B (aa2238-2465). Interaction was assessed by yeast growth on SD-L/-T/-H/-A medium. Empty vectors were used as negative controls, SV40 large T antigen and p53 were used as positive controls. (b) Nbr1 is found in a complex with MAP1B-LC1. Coimmunoprecipitation of HA-Nbr1 and MAP1B-LC1-myc from COS7 cells transfected with HA-Nbr1 and MAP1B-LC1-myc constructs. Extracts and precipitates were analysed by western blot using the indicated antibodies.

To identify novel protein interactors of the highly conserved FW domain of Nbr1 and therefore elucidate a function, we performed a yeast-2-hybrid screen with the FW domain of Nbr1 as bait. A neonatal calvarial cDNA library was screened and the light chain of the microtubule-associated protein 1B (MAP1B-LC1) was identified as an interaction partner of Nbr1. This interaction was verified by a directed yeast two-hybrid assay by retransforming the isolated prey vector encoding the partial MAP1B-LC1 sequence (aa2238-2465) into yeast strain AH109 and mating it with yeast strain Y187 that was expressing the FW domain of Nbr1 (Figure 3(a)). MAP1B is transcribed as a single mRNA, translated into a polypeptide, and subsequently cleaved producing a heavy chain (2214aa) and a light chain (250aa) [38]. Both the heavy chain (MAP1B HC) and the light chain (MAP1B-LC1) can bind to microtubules [39, 40] and to each other [41]. MAP1B has been implicated in the regulation of autophagy, as it interacts with LC3 and targets autophagosomes to axon terminals during neurodegeneration [42].

### 3.3. Nbr1 is Found in a Complex with MAP1B-LC1 *In Vivo*

To determine whether Nbr1 forms a complex with MAP1B-LC1 *in vivo*, we performed a coimmunoprecipitation experiment using COS7 cells transiently transfected with HA-Nbr1 and MAP1B-LC1-myc constructs. Using an anti-myc antibody for immunoprecipitation of MAP1B-LC1-myc, we found that HA-Nbr1 did coimmunoprecipitate with MAP1B-LC1-myc (Figure 3(b)) confirming that they are found in a complex *in vivo*. We were unable to show coimmunoprecipitation of endogenous Nbr1 and MAP1B-LC1 in PC12 cells. This is likely to be due to the levels of interacting protein being below the detection level possible by western blot analysis with the available antibodies (data not shown).

### 3.4. Nbr1 Interacts with MAP1B-LC1 *In Vitro*

**Figure 4:**
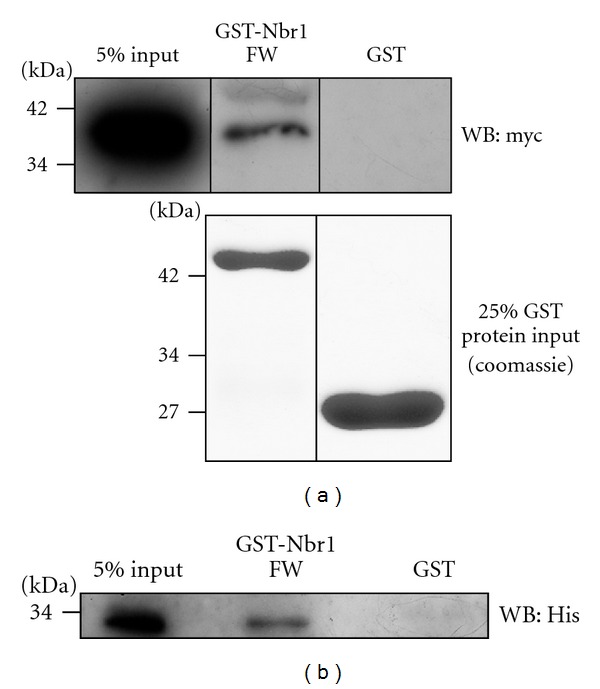
Nbr1 interacts with MAP1B-LC1. (a) GST pulldown assay using cell extracts from COS7 cells transfected with MAP1B-LC1-myc and immobilised GST or GST-Nbr1 FW domain. Upper panel: coprecipitated proteins were detected with an anti-myc antibody. The FW domain of Nbr1 interacts with MAP1B-LC1. Lower panel: coomassie stained SDS PAGE gel showing 25% of GST-tagged protein input. (b) GST pulldown assay using purified His-MAP1B-LC1 and immobilised GST or GST-Nbr1 FW domain. Coprecipitated proteins were detected using an anti-His antibody and demonstrated that the FW domain of Nbr1 interacts with MAP1B-LC1. The same amount of GST or GST-Nbr1 FW domain fusion protein was used as shown in (a) (lower panel).

The yeast-2-hybrid data suggested that the FW domain of Nbr1 interacts directly with MAP1B-LC1. To more rigorously test this hypothesis, we performed GST pull-down assays using extracts from COS7 cells overexpressing MAP1B-LC1-myc and a GST fusion of the FW domain of Nbr1 and GST alone. Indeed, the FW domain of Nbr1 interacted with MAP1B-LC1 whilst GST alone did not (Figure 4(a)).

To verify the interaction between the FW domain of Nbr1 and MAP1B-LC1 in a cell-free environment, His_6_-MAP1B-LC1 was purified and incubated with the GST fusions of the FW domain of Nbr1 or GST alone. This demonstrated that the FW domain of Nbr1 interacts directly with the light chain of MAP1B (Figure 4(b)).

### 3.5. MAP1B Is Not Degraded by Autophagy

**Figure 5:**
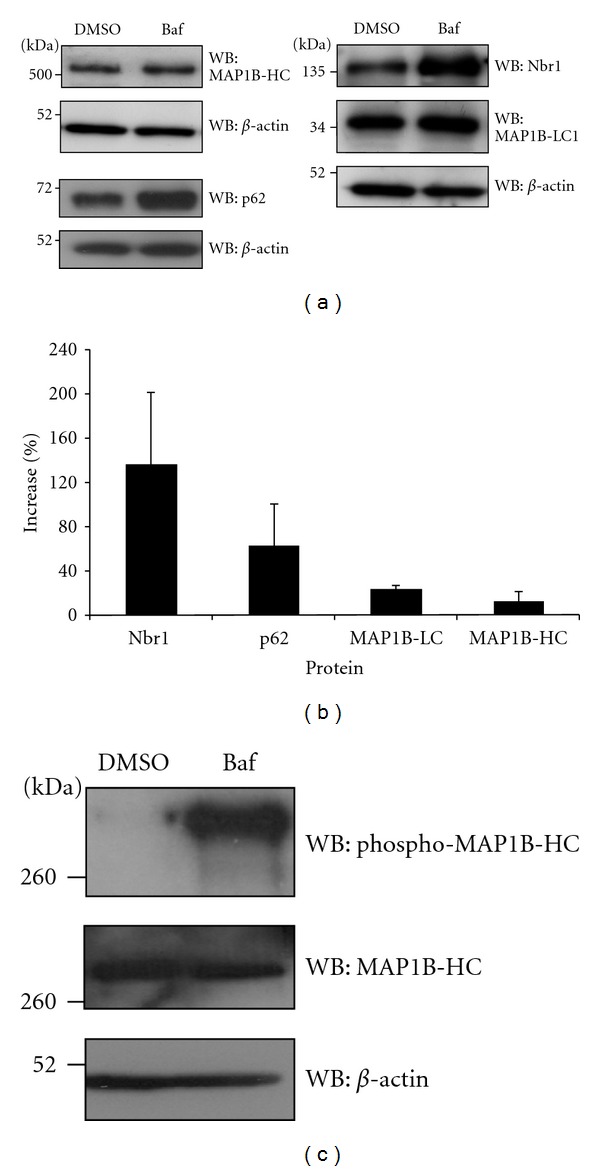
Western blot analysis of p62, Nbr1, MAP1B-HC, and MAP1B-LC1 protein levels following blockage of autophagic protein degradation. (a) Western blots showing protein levels in cells treated with DMSO (control) or Bafilomycin A1 (Baf). (b) Quantification of protein band intensity. MAP1B-LC1 and MAP1B-HC levels are increased by a negligable amount compared with Nbr1 and p62 upon treatment with Bafilomycin A1; Error bars represent SD, *n* = 3. (c) Phospho-MAP1B-HC is degraded by autophagy. Upon blockage of autophagic degradation with Bafilomycin A1: (Baf), levels of phospho-MAP1B-HC increase compared with levels of total MAP1B-HC.

It has previously been observed that MAP1B-HC is not degraded by autophagy [42] however, it has not been reported whether the same is true for MAP1B-LC1. To establish if the function of the interaction between Nbr1 and MAP1B-LC1 is to facilitate the degradation of MAP1B-LC1 via autophagy, MAP1B-LC1 protein levels were analysed under conditions where autophagic protein degradation was blocked. PC12 cells, a neuronal cell line that expresses elevated levels of endogenous MAP1B, were treated with Bafilomycin A1 or DMSO for 8 hours before protein extracts were resolved by SDS PAGE and detected using antibodies that recognise p62, Nbr1, MAP1B-LC1, MAP1B-HC and *β*-actin. Upon blockage of autophagic protein turnover, the levels of p62 and Nbr1 were increased by 60% and 130%, respectively, demonstrating that autophagic protein degradation was blocked by Bafilomycin A1 treatment (Figures 5(a) and 5(b)). MAP1B-HC, and MAP1B-LC1 protein levels showed a negligible increase upon the blockage of autophagic protein degradation suggesting that they are not degraded by autophagy and that the function of the Nbr1-MAP1B-LC1 interaction is not to target MAP1B-LC1 for autophagic protein turnover. Surprisingly, although total MAP1B levels are largely unaffected by blocking autophagic protein degradation, levels of phospho-pThr1265-MAP1B are increased following Bafilomycin A1 treatment (Figure 5(c)). This phosphorylated form of MAP1B is expressed in differentiating neurons and is a major substrate for glycogen synthase kinase-3beta (GSK-3beta) and is thought to be involved in regulating microtubule dynamics by MAP1B [43].

### 3.6. Nbr1 Colocalises with MAP1B upon Induction of Autophagy

**Figure 6:**
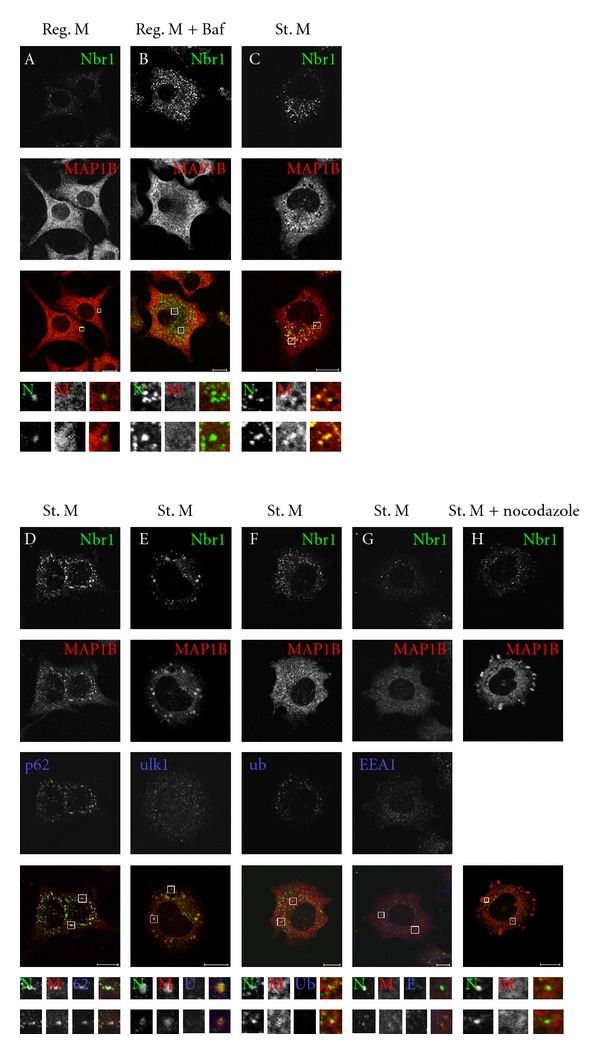
Nbr1 and MAP1B colocalise in discrete perinuclear vesicles upon induction of autophagy. PC12 cells were treated with DMSO, Bafilomycin or starved then fixed and stained with antibodies against the indicated proteins. Under basal conditions (A) or when autophagic degradation is blocked by Bafilomycin A1 treatment (B), very little or no colocalisation was observed between Nbr1 and MAP1B. When cells were starved to induce autophagy (C) MAP1B and Nbr1 colocalise in distinct perinuclear vesicles which are also positive for p62 (D) but are largely negative for ULK1 (E), ubiquitin (F), and EEA1 (G). Upon depolymerisation of the microtubule network and subsequent induction of autophagy by starvation, MAP1B no longer colocalised in distinct perinuclear vesicles with Nbr1 (H). Antibodies used: anti-Nbr1 (abcam), anti-MAP1B (N19, Santa Cruz), anti-p62 (M. Gautel, KCL), anti-ULKl (Sigma), anti-ubiquitin (Ub) (Sigma), and anti-EEAl (Cell Signaling). Scale bar; 10 *μ*m.

Next, we analysed the subcellular localisation of endogenous Nbr1 and MAP1B by confocal microscopy. To establish if Nbr1 and MAP1B colocalise *in vivo*, PC12 cells were treated with DMSO, Bafilomycin A1 to block autophagic protein degradation or starved to induce autophagy and analysed by immunofluorescence. Under basal conditions, when levels of Nbr1 are low, there was little colocalisation between Nbr1 and MAP1B (Figure 6(A)). Upon blockage of autolysosomal protein degradation by Bafilomycin A1 treatment, Nbr1 is no longer turned over by autophagy and accumulates (Figure 6(B)) but total MAP1B is unaffected and appears excluded from Nbr1-positive vesicles. This confirms that MAP1B is not itself degraded by the autolysosomal pathway. Upon starvation and induction of autophagy, Nbr1 and MAP1B colocalise to distinct perinuclear vesicular structures (Figure 6(C)). Although this does not occur in all cells, only under starvation conditions were MAP1B-/Nbr1-positive vesicles observed. Under starvation conditions where MAP1B/Nbr1 positive punctate structures were observed, quantification of colocalisation showed a Mander's colocalisation coefficient of 64 ± 10%. These MAP1B-/Nbr1-positive vesicles also colocalise with the autophagic protein p62 (Figure 6(D)) but few colocalise with ubiquitin, suggesting that these vesicles are not aggresomes or mature autophagosomes loaded with ubiquitinated cargo (Figure 6(F)). We found little overlapping distribution with ULK1 and Nbr1/MAP1B vesicles under starvation conditions, in comparison with previous analysis of Nbr1/ULK1 colocalisation under these conditions [32] (Figure 6(E)) or with the early endosomal marker EEA1 (Figure 6(G)). This demonstrates that upon induction of autophagy, Nbr1 is recruited to MAP1B positive structures which are colocalising with p62, suggesting these may be early autophagosomes but downstream of autophagosomal formation sites.

To determine if colocalisation of Nbr1 and MAP1B in response to starvation-induced autophagy was dependent upon an intact microtubule network, PC12 cells were treated with the depolymerisation agent nocodazole under starvation conditions and examined for colocalisation. Depolymerisation of the microtubule network was confirmed by *α*-tubulin staining (data not shown) and resulted in loss of the punctate colocalisation of MAP1B and Nbr1 but intact Nbr1 vesicles were retained (Figure 6(H)). This suggests that MAP1B is not essential for the formation of Nbr1-positive vesicles but that an intact microtubule network is essential for colocalisation of Nbr1 and MAP1B under starvation conditions.

## 4. Discussion

The FW domain of Nbr1 is highly conserved throughout the eukaryotic kingdom and is also present in a number of bacterial proteins. It contains two internal repeats of ~55 residues and has a predicted secondary structure consisting of two, three *β*-stranded sheets. The high conservation of this region and its absence in p62 [37] suggests that it has a function that is distinct from p62. We therefore performed a yeast-2-hybrid screen with the FW domain of Nbr1 in order to determine a specific function for this region. The light chain of MAP1B (MAP1B-LC1) was identified as an interaction partner of the FW domain. As Nbr1 has previously been identified as an autophagic receptor that targets ubiquitinated proteins for degradation via its interaction with LC3 [18, 19], it was reasonable to hypothesise that the function of the interaction between Nbr1 and MAP1B-LC1 is to facilitate the autophagic degradation of MAP1B-LC1. Analysis of protein levels after autophagy blockage demonstrated that the levels of MAP1B-LC1 increased by a negligible amount suggesting that it is not degraded by autophagy (Figure 5). Blockage of autophagosomal protein degradation can also result in a reduction of protein turnover by the UPS [44] therefore, as MAP1B-LC1 is known to be degraded by the UPS [45], this could suggest that Bafilomycin A1 treatment results in the inhibition of MAP1B-LC1 degradation via the proteasome rather than by autophagy. Interestingly, we observed that inhibition of autophagic degradation resulted in an increase in phospho-Thr1265 MAP1B, perhaps also reflected in the small increase in total MAP1B levels observed. Expression of this phosphorylated form of MAP1B is spatially regulated in differentiating neurons, and the kinase responsible for phosphorylation at this site has been identified as glycogen synthase kinase-3 beta. GSK-3 beta inhibition has been linked to Bif-1-dependent autophagic induction under serum starvation to modulate cell survival [46].

Further biochemical analysis confirmed that the interaction between Nbr1 and MAP1B-LC1 is direct and that these proteins can be found in a complex together *in vivo. *As both Nbr1 and the microtubule network have been identified as key players in the facilitation of protein degradation via autophagy [11, 12, 18, 19], this could suggest that the Nbr1-MAP1B-LC1 interaction is important for this process. MAP1B interacts with LC3 and through this interaction it has been proposed that autophagosomes are targeted to axon terminals during neurodegeneration [47]. Additionally MAP1B has been predicted to interact with Atg12 and Atg3 suggesting that in addition to LC3, MAP1B is important for targeting other components of the autophagosomal machinery to sites of autophagosomal formation [48]. The interaction data presented here and the colocalisation of Nbr1 and MAP1B to perinuclear vesicles suggest that via its interaction with MAP1B, Nbr1 is targeted to the microtubule network, thus providing a mechanism by which proteins can be targeted to autophagosomes. The MAP1B-/Nbr1-positive vesicles do not however colocalise with ubiquitin, suggesting that these vesicles are not yet loaded with ubiquitinated cargo. Alternatively, they could represent vesicles loaded with other nonubiquitinated proteins that have been targeted for degradation. Whilst there are currently no known proteins that are targeted for autophagy by Nbr1 in a ubiquitin-independent manner, STAT5A-ΔE18 can be targeted for autophagic degradation by the PB1 domain of p62 independent of ubiquitin [49]. This suggests that Nbr1 could also be acting by a similar mechanism to target proteins for degradation independent of ubiquitin. Nbr1/MAP1B vesicles did not colocalise with EEA1, showing that these are not early endosomes. Likewise, we saw largely no colocalisation of MAP1B/Nbr1 vesicles with ULK1, suggesting that MAP1B- and Nbr1-positive structures are not present at sites of autophagosomal formation but do perhaps represent early autophagosomes that are positive for p62 and nonubiquitinated protein cargo.

This is the first evidence linking Nbr1 to the microtubule network and also demonstrates a distinct function for the FW domain of Nbr1. A similar mechanism has previously been demonstrated whereby HDAC6 is able to interact with polyubiquitinated protein aggregates and to dynein motors thereby coupling protein aggregates to the microtubule network where they can be transported to sites of autophagosomal formation [50]. Furthermore, adaptor proteins such as FYCO can interact with LC3 and microtubule motor proteins and through these interactions it has been suggested that preautophagosomal membranes are targeted to sites of autophagosomal formation [51]. Roles for MAP1S (a MAP1B homologue) in autophagic degradation of mitochondria have also been demonstrated. MAP1S interacts with LC3 and this interaction functions to target LC3, to the microtubule network. Genetic ablation of MAP1S causes the accumulation of defective mitochondria and severe defects in response to nutritive stress suggesting defects in autophagosomal biogenesis and clearance [52]. It has been suggested that recruitment of autophagosomal cargo receptors like Nbr1 and p62 to the autophagosomal formation site may be a general feature of this type of receptor, but that it is independent of Atg factors downstream of the PI3-kinase complex [32]. This study further highlights the role for microtubule associated proteins in the targeting of autophagosome machinery to the microtubule network and complements the work presented here that suggests a link between microtubule-associated proteins and autophagic receptors.

The high evolutionary conservation of the FW domain within Nbr1 homologues implicates it to have a critical role in Nbr1 function. The predicted secondary structure of the FW domain that consists of two three *β*-stranded sheets that form a compact “sandwich” is also present in the cholesterol-binding protein Niemann-Pick C2 (NPC2) [53, 54] suggesting additional roles for the FW domain in lipid binding.

In summary, we present the first evidence linking the autophagic receptor protein Nbr1 and the microtubule network via a direct interaction of the evolutionary-conserved FW domain of Nbr1 with MAP1B. Nbr1 is a ubiquitously expressed protein that has been implicated in several diseases [18, 22, 23], and it therefore will be of significant value to assess this interaction in tissue-specific physiological studies.
